# Black Salve (or escharotics). A picture about an unorthodox medical preparation in oncology

**DOI:** 10.1002/ski2.241

**Published:** 2023-05-09

**Authors:** Yannick Borkens

**Affiliations:** ^1^ Charité Universitätsmedizin Berlin Berlin Germany; ^2^ Humboldt‐Universität zu Berlin Berlin Germany

## Abstract

Alternative medicine methods and treatments enjoy great popularity. However, many users fail to recognise the dangers associated with these methods. One of these is called Black Ointment or Black Salve. Oncologists and attending physicians should be aware of alternative medical methods such as these and be in a position to provide appropriate advice, intervention, if necessary, at any time when counselling patients in difficult situations.

## Graphical abstract

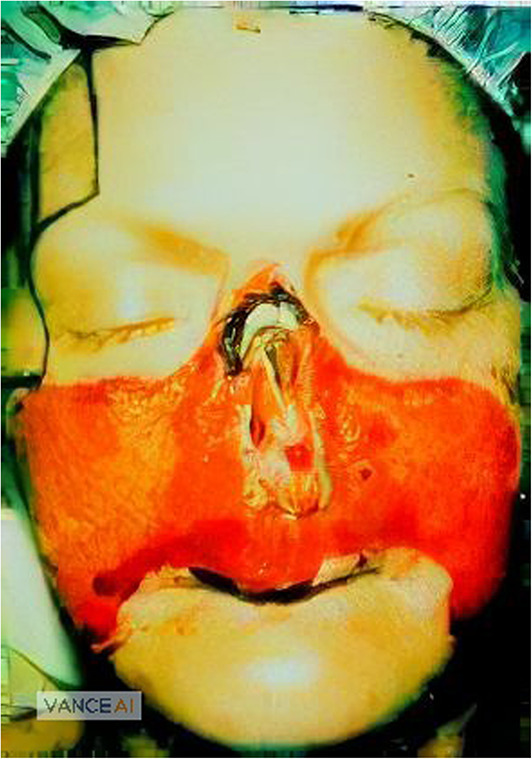

## 1 INTRODUCTION

Alternative healing methods are enjoying some popularity. Not least due to the SARS‐CoV‐2 pandemic, these methods have recently experienced a real boom. Thus, in the case of SARS‐CoV‐2, various products are being touted as cures and medicines.^1^ Among other things, this current boom led to the current pandemic being referred to as an infodemic.^2^, ^3^ For patients, but also for society in general, this creates relevant dangers. However, so‐called alternative and *natural* medicines are not only used for infectious diseases. In fact, oncology is one of the most important areas of alternative medicine.^4^, ^5^ The prevalence of these methods may be related, among other things, to the finality of the diagnosis of cancer. Especially in countries of the Global North, where due to the medical infrastructure infectious diseases do not have the same relevance as in countries of the Global South, cancer as one of the most relevant causes of death plays an important role in the health care systems and in the general perception. Overall, cancer represents the highest clinical, social, and economic burden in terms of cause‐specific *Disability‐Adjusted Life Years* (DALYs) among all diseases. With approximately 8.97 million deaths per year and an overall risk of 20.2% (22.4% for men, 18.2% for women) for cancer in people aged 0–74 years, cancer is the second leading cause of death, after ischaemic heart disease.^6^ The most common cancer types include lung, breast and prostate cancer. By 2060, cancer will likely displace heart disease from first place. Scientists and physicians then expect up to 18.63 million deaths annually.^6^, ^7^ The psychological factors of cancer should not be ignored either. Many patients also suffer from depression after their cancer diagnosis and quite a few lose their courage and will to live.^8^, ^9^ It is therefore not surprising that sellers and distributors of alternative medicine methods and preparations take advantage of this concern of patients. The methods and preparations offered are as diverse as they are comprehensive.

Besides the classical and widely used methods such as homoeopathy^10^, ^11^ or traditional Chinese medicine,^12^ there are also more exotic and lesser‐known methods. These include, for example, the use of various herbs and medicinal plants,^13^, ^14^ Ayurveda,^15^ Qigong,^16^ or frog poisons called Kambô.^17^ The evidence and effectiveness of these methods are often scientifically untenable. For example, an effectiveness of homoeopathy that goes beyond the placebo effect and usual context effects has still not been proven.^18^, ^19^ In some cases, the applied methods even have a strongly damaging effect that additionally endangers the patient. One of these methods is discussed in this article. The black salve (or black salves, as there are different ointments with different formulations; another name is escharotics) is also touted by esotericists as a cure for cancer. The salves, which are also called and advertised as anti‐cancer ointments, are applied to the skin and are supposed to *etch* or *draw* the cancer out of the body. Primarily, they are used in the treatment of skin cancer, to which the ointment is to be applied directly. However, various alternative medicine practitioners also promote their use for deeper cancers (breast cancer is frequently mentioned. Moreover, in 2018, a woman died in the U.S. trying to cure her ovarian cancer with a black salve).

**FIGURE 1:**
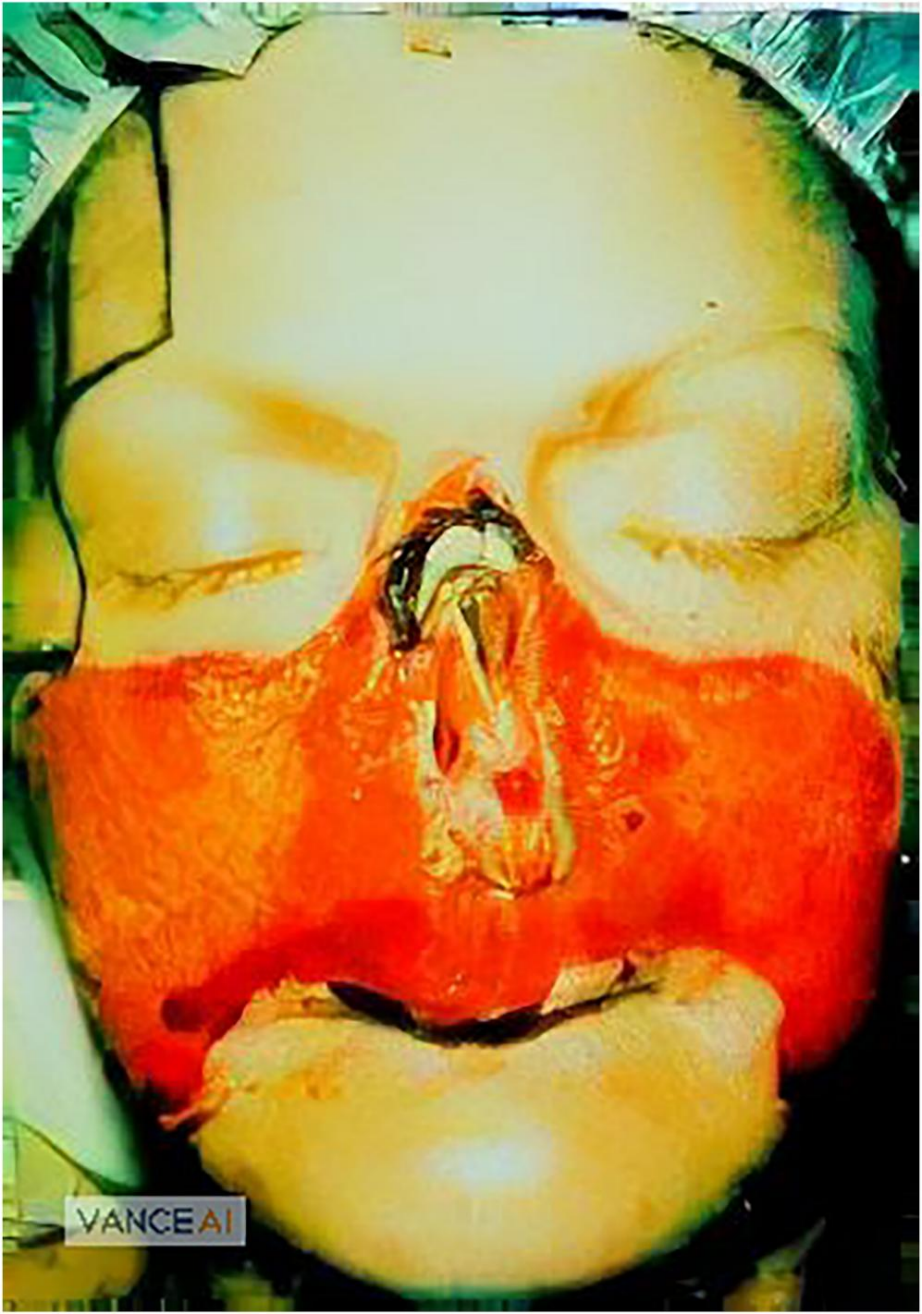
Case R. Conrad.

**FIGURE 2:**
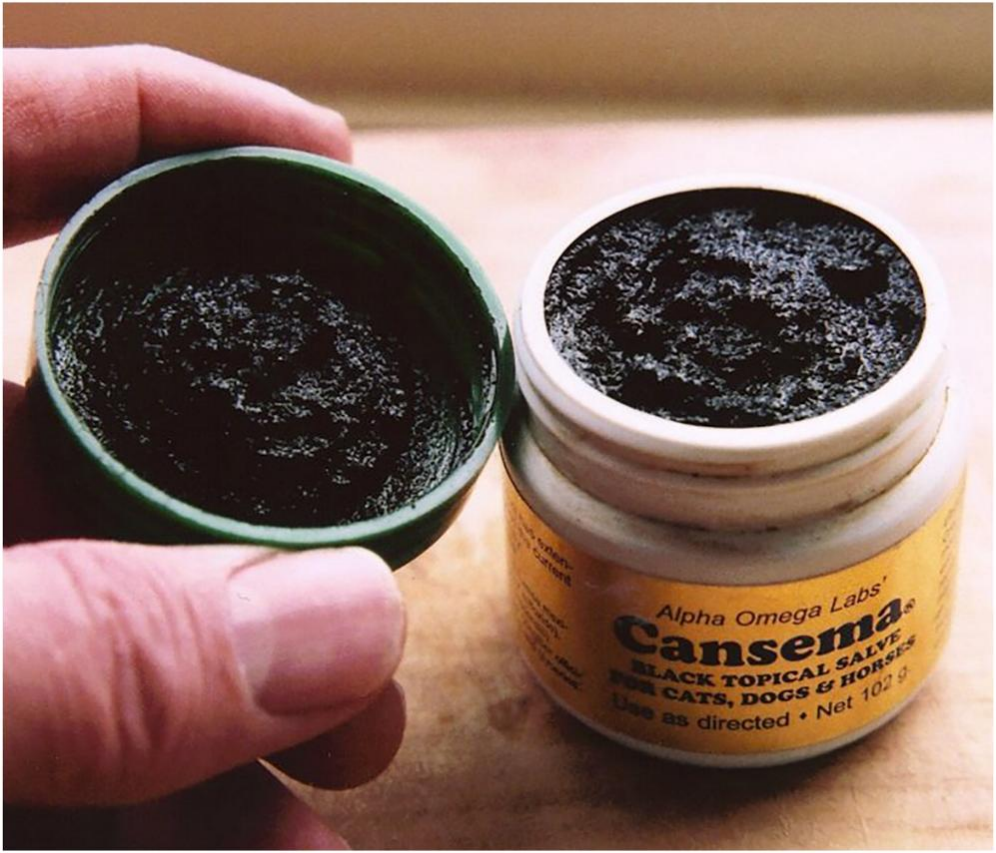
Black Salve. Shown here is the product Cansema from the manufacturer Alpha Omega Labs from Ecuador. On its product website, this company advertise with the slogan *The Triumph of Medical Science Over‐Politics and Greed*.

Because the ointment itself is highly corrosive, patients are at risk of scarring and skin burns in the “best” case, and severe mutilation in the worst case. Figure 1 shows such a mutilation. The case of R. Conrad occurred in Idaho in 1984. The patient was not suffering from cancer, but from a simple shoulder pain. With this she visited a local naturopath. During the examination, she also referred to a bump on her nose, which the Naturopath diagnosed as cancer. To treat this “cancer,” he gave her a black salve that Conrad was to apply to the bump. Within a very short time, she described severe facial pain, a development of heat, and red streaks. The naturopath, whom she contacted again about the complaints, placated her, presenting the injuries as an initial aggravation. Then, within a week, the injuries and mutilations appeared as shown in the figure. The naturopath later described getting the ointment from Mexico and not knowing the manufacturer or the exact origin. Conrad's face could be restored. However, this process took 3 years and required 17 plastic surgeries.^20^ In 2020, the U.S. FDA stated to have documented 24 such cases in recent years, 15 of them in the last 7 years^21^ Although these figures give the impression that preparations such as black salve are a recent modern phenomenon, anti‐cancer ointments have been in use for many years. Such ointments were used as early as the 18th and 19th centuries. Besides cancer, other diseases were also treated with these ointments, for example, herpes infections. The name black salve is no coincidence, as Figure 2 shows. The black colouration is due to activated charcoal, which is used in various (but not all) preparations.

## 2 INGREDIENTS

As already described, there are various preparations, which are called black salves. The composition is always slightly different. Thus, not all ointments contain activated charcoal. But what can be found in every ointment is zinc chloride (ZnCl_2_). Zinc chloride can be called the main ingredient for this reason. In addition, there are various medicinal plants. Canadian bloodroot (*Sanguinaria canadensis*) is widely used. Ribwort plantain (*Plantago lanceolata*) can also be used as an ingredient. Another ingredient is dimethyl sulfoxide (DMSO), an organic solvent. DMSO is used in pharmacy as a percutaneous drug, for example, in the treatment of local pain, bruising, or sports injuries.^22^, ^23^, ^24^

**FIGURE 3:**
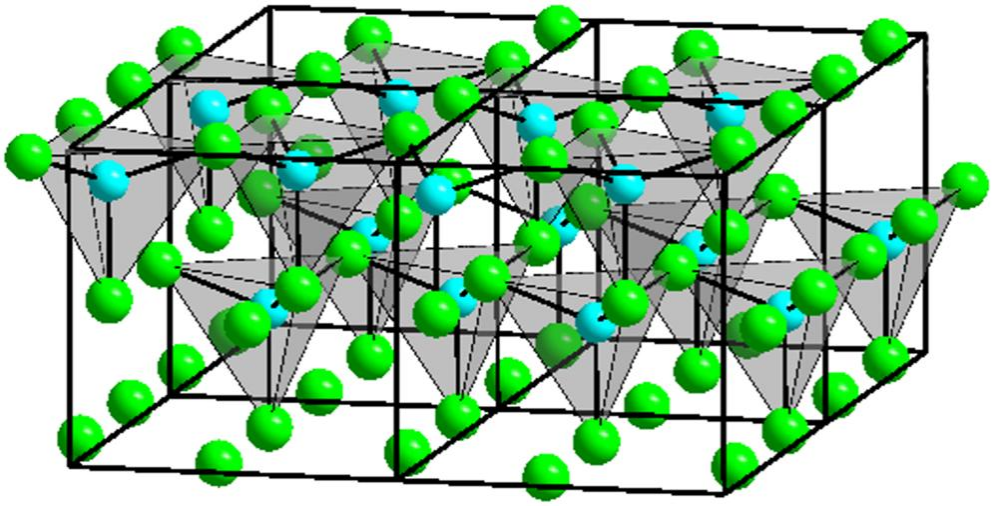
Crystal structure of *α*‐zinc chloride (ZnCl_2_). Zn^2+^ is shown in blue, Cl^−^ is shown in green.

**FIGURE 4:**
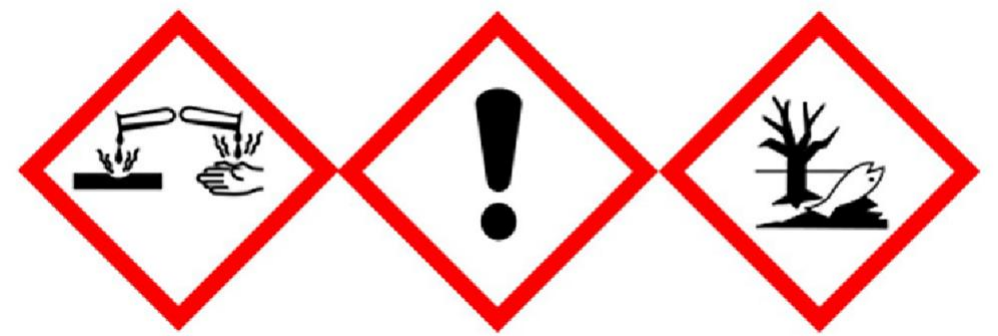
*GHS‐Gefahrstoffkennzeichnung* of zinc chloride. *GHS‐Gefahrstoffkennzeichnungen* are used in Germany to label hazardous chemicals. Different labels identify different properties. The properties shown here are *corrosive* (left), *harmful* (middle) and *harmful to the environment* (right).

As the main component, zinc chloride should be considered in more detail. Zinc chloride is a crystalline compound, formed when zinc (Zn^2+^) is heated in chlorine (Cl^−^) or when zinc sulfate (ZnSO_4_) is heated with calcium chloride (CaCl_2_). It appears as a white, granular powder consisting of hexagonal‐rhombohedral flakes. Figure 3 shows the crystal structure. The chemical is highly corrosive and capable of dissolving tissues such as plant fibres. In addition, it removes water from organic matter. It has a burning taste, is corrosive when swallowed and a strong irritant when inhaled.^25^ The LD_50_ in rats (oral) is 350 mg/kg.^26^ Figure 4 shows the German *GHS‐Gefahrstoffkennzeichnungen* associated with zinc chloride. On the skin, zinc chloride causes ulceration^27^ and burns.^28^, ^29^ Possible contact dermatitis has also been reported.^25^ For ZnCl_2_, neither carcinogenicity nor reproductive toxicity can be proven beyond doubt. However, these cannot be completely ruled out either.^30^, ^31^, ^32^ In the industry, it is used for the impregnation of wood, in pickling as well as in disinfecting.^33^, ^34^ It also gets important medical relevance as a component of smoke bombs, the use of which can lead to pneumonia and poisoning.^35^, ^36^

**FIGURE 5:**
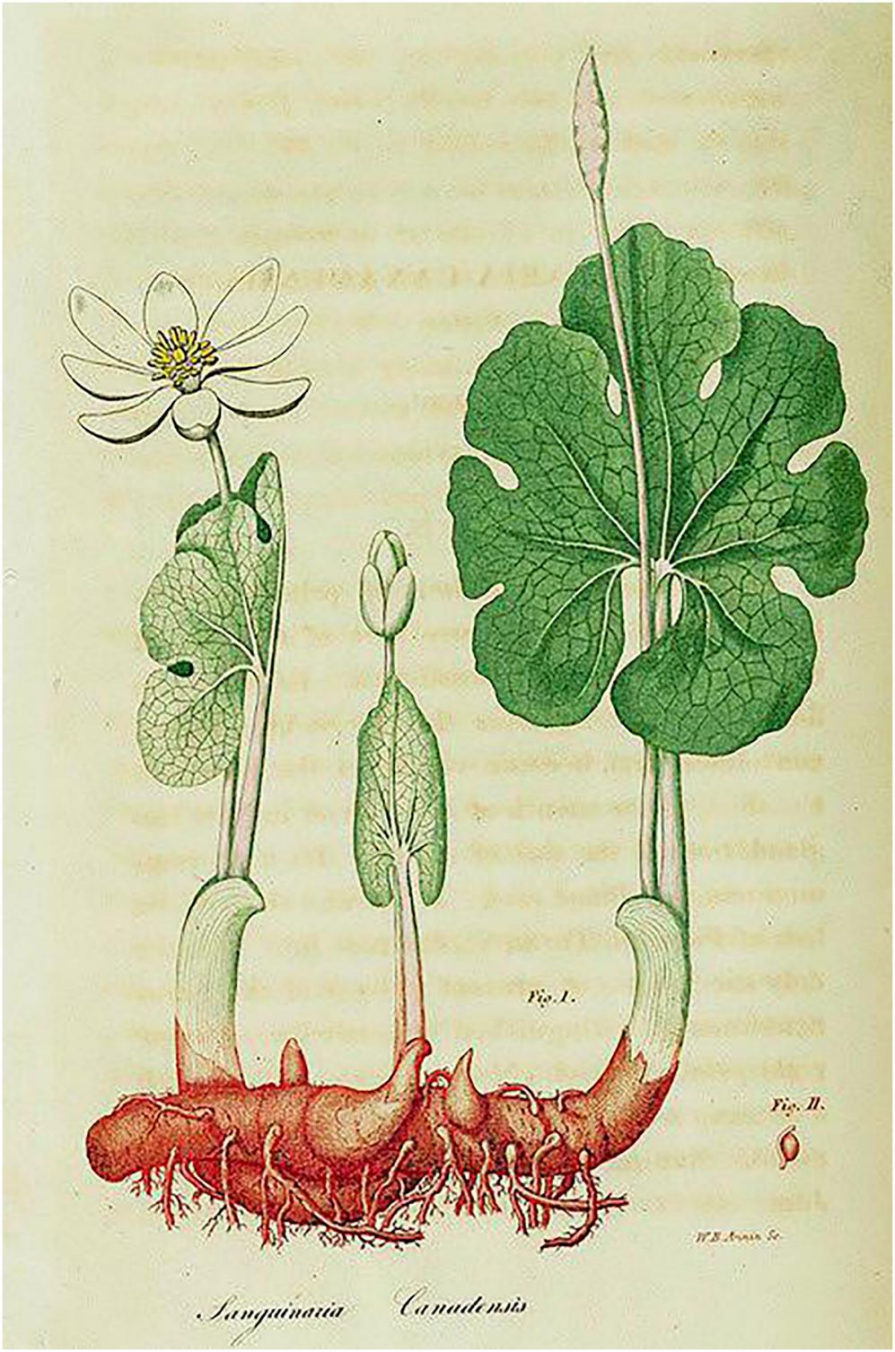
Canadian bloodroot (*Sanguinaria canadensis*). Botanical drawing.

In addition to zinc chloride, various medicinal plants are used. Since different recipes mention different medicinal plants, only Canadian bloodroot is discussed here. Canadian bloodroot (*Sanguinaria canadensis*) is a member of the poppy family (Papaveraceae), which includes the well‐known poppies such as the corn poppy (*Papaver rhoeas*) and comprises 41 genera with a total of 800 species.^37^, ^38^ *Sanguinaria canadensis* is the only species of the genus *Sanguinaria*. There is no relationship with bloodroot (*Potentilla erecta*, *P*. *erecta* belongs to the rose family [Rosaceae]). The name Sanguinaria is derived from the Latin word sanguineus for blood. Figure 5 shows a botanical drawing of *S*. *canadensis*. Its ecological distribution is wide, from eastern Canada through the Great Lakes region and into the southern United States (including both Florida and Texas). The plant can be found at elevations ranging from 0 to 1200 m above sea level.^39^ Already the indigenous inhabitants of America used the Canadian bloodroot to treat several conditions like fever or rheumatism or to induce vomiting specifically. *S*. *canadensis* is said to have certain antimicrobial, antihypertensive and anti‐inflammatory effects. In homoeopathy, the plant or sanguinarine, which is found in the root, is used for preparations according to homoeopathic principles to promote expectoration of phlegm or to relieve sore throat. Homoeopaths also resort to bloodroot for female menopause.^40^, ^41^ To date, however, its effects have not been substantiated, nor have pharmaceutical agents been developed from its constituents (such as sanguinarin).^42^ The sap can cause tissue damage when applied to the skin.^43^

Nevertheless, bloodroot is experiencing a renaissance, especially recently, in the treatment of various skin diseases, including skin cancer. These treatments do not necessarily have to be related to black salve. There is a wide range of different skin products that contain bloodroot and are used to treat various ailments. Dermatologists believe that a possible reason is the Internet, which makes it much easier to market those various products.^44^, ^45^

## 3 CONCLUSION FOR PRACTICE

The black salve presented here is just one example of how dangerous alternative medicine (commonly referred to as ‘soft and gently’ medicine) can be. In the case of anticancer ointments, there is a direct danger from the treatment to the patients. Two factors should be considered: First, the purely organic‐medical ones, and second, the psychological ones. The organic‐medical ones include the mutilations and burns. In this case, the skin cancer itself is not attacked. While the Black Ointment cauterises the surface of the body, the cancer continues to grow in deeper layers without being affected by the ointment. Accordingly, metastases are common in some skin cancer types like malignant melanoma and can affect visceral tissues like the brain, lung or liver (distant metastases—not in the skin).^46^, ^47^ They are more critical for the course of the disease, but not affected by the ointment. Also, the psychological aspects should not be disregarded. In addition to the stress caused by the cancer, there are now those resulting from the mutilation caused by the treatment.^48^, ^49^, ^50^ Oncologists and attending physicians should be aware of alternative medical methods such as these and be in a position to provide appropriate advice, intervention, if necessary, at any time when counselling patients in difficult situations. Experience has shown that a large proportion of cancer patients ‐ latent and manifest ‐ are inclined towards “alternative” methods, at least as an adjunctive therapy. What is needed here is the establishment of a positive doctor‐patient relationship and confidence‐inspiring counselling to get patients to disclose whether and what concomitant medications they are using to avert danger (as in black salve) and unnecessary suffering. In a Yale University study that correlated relevant compliance impairments with complementary use of “alternative” concomitant therapies, the authors recommend that patients always be made aware of the risks of such agents and methods.^51^

## CONFLICT OF INTEREST STATEMENT

None to declare.

## AUTHOR CONTRIBUTIONS

**Yannick Borkens**: Conceptualization (Equal); Data curation (Equal); Formal analysis (Equal); Investigation (Equal); Methodology (Equal); Validation (Equal); Visualization (Equal); Writing – original draft (Equal); Writing – review & editing (Equal)

## ETHICS STATEMENT

Not applicable.

## Data Availability

In this article no data generated.
